# Supplementary material for: Safety, Feasibility and Technical Considerations from a Prospective, Observational Study—CIREL: Irinotecan-TACE for CRLM in 152 Patients
Source: J Clin Med. 2022 Oct 19;11(20):6178. doi: 10.3390/jcm11206178 (PMC9604674; doi:10.3390/jcm11206178)
Supplement: Supplementary file 1 [file jcm-11-06178-s001.zip › jcm-1947440-supplementary.pdf]

Supplementary Table S1.

	LLN	ULN	UNIT	G1	G2	G3	G4	UNIT
Lymphocytes	1.2		x 10^9/L	< LLN	< 0.8	< 0.5	< 0.2	x 10^9/L
Platelets	150		x 10^9/L	< 150	<75	< 50	< 25	x 10^9/L
Neutrophils	2		x 10^9/L	< 2	< 1.5	< 1	< 0.5	x 10^9/L
Alkaline phosphatase		120	U/L	> ULN >120	> ULN x 2.5 >300	> ULN x 5 >600	> ULN x 20 >2400	U/L
Albumin	3.5		g/dL	< 3.5	< 3	< 2		g/dL
AST /SGOT		40	U/L	> ULN 40	> ULN x 3 120	> ULN x 5 200	> ULN x 20 800	U/L
ALT /SGPT		40	U/L	> ULN 40	> ULN x 3 120	> ULN x 5 200	> ULN x 20 800	U/L
Blood Bilirubin		1	mg/dL	> ULN 1	> ULN x 1.5 1,5	> ULN x 3 3	> ULN x 10 10	mg/dL
Serum Creatinine		1.3	mg/dL	> ULN 1.3	> ULN x 1.5 1.95	> ULN x 3 3.9	> ULN x6 7.8	mg/dL
LDH		225	U/L	>ULN				U/L

Abnormal laboratory grading scheme according to CTCAE 4.03 and CTCAE 5.0 (for LDH). Reference values and the resulting CTCAE-based cut-offs for grading abnormal laboratory values. Red values are based on ULN/LLN and therefore depend on the selected reference ranges. Source for reference values: https://www.abim.org/~/media/ABIM%20Public/Files/pdf/exam/laboratory-reference-ranges.pdf.

Supplementary Table S2. Association of patient and treatment-related factors and AEs. Data is presented as counts and percentages. Significant differences between categorical data were assessed using Chi-squared test to compare patients with ≥1 AEs and patients with 0 AEs (*p* values ≤ 0.05 were considered significant).

	**Peri-interventional AEs (Day 1) N = 152 Patients**		**Acute AEs (Day 2 – Day 30) N = 152 Patients**	
	**0 AEs**	**≥1 AEs**	**0 AEs**	**≥1 AEs**
Treatment intention, n (%)				
First line treatment or consolidation therapy after response to first line	16 (39)	25 (61)	27 (66)	14 (34)
Combination treatment with ablation with a curative intent	9 (47)	10 (53)	14 (74)	5 (26)
Intensification of treatment with/without concomitant therapy	10 (24)	31 (76)	29 (71)	12 (29)
Salvage treatment in progressive patients	24 (52)	22 (48)	31 (67)	15 (33)
Other	2 (40)	3 (60)	2 (40)	3 (60)
	p-value: 0.1		*p*-value: 0.68	
Performance status (ECOG) n (%)				
0	42 (47)	47 (53)	73 (82)	16 (18)
1	15 (29)	37 (71)	25 (48)	27 (52)
2	3 (30)	7 (70)	4 (40)	6 (60)
3	1 (100)	0 (0)	1 (100)	0 (0)
	p-value	0.09	*p*-value	<0.001
% of liver involvement, n (%)				
< 25%	44 (54)	38 (46)	57 (70)	25 (30)
25 – 50%	14 (24)	45 (76)	36 (61)	23 (39)
> 50%	3 (27)	8 (73)	10 (91)	1 (9)
	p-value	0.001	*p* value	0.13
Extrahe*p*atic metastases, n (%)				
No	39 (45)	47 (55)	62 (72)	24 (28)
Yes	22 (33)	44 (67)	41 (62)	25 (38)
	p-value	0.18	*p*-value	0.26
Previous lines of liver metastases directed systemic therapy, n (%)				
no lines	11 (46)	13 (54)	18 (75)	6 (25)
1-2 lines	41 (37)	70 (63)	79 (71)	32 (29)
3 or more lines	8 (50)	8 (50)	7 (44)	9 (56)
	p-value	0.49	*p*-value	0.07
Previous use of irinotecan, n (%)				
No	23 (37)	39 (63)	41 (66)	21 (34)
Yes	38 (43)	51 (61)	61 (69)	28 (32)
	p-value	0.56	*p*-value	0.34
Previous intra-arterial treatment received, n (%)				
No	55 (41)	80 (59)	93 (69)	42 (31)
Yes	6 (35)	11 (65)	10 (59)	7 (41)
	p-value	0.86	*p*-value	0.57
Previous ablation on liver metastases performed, n (%)				
No	53 (39)	82 (61)	91 (67)	44 (33)
Yes	8 (47)	9 (53)	12 (71)	5 (29)

	p-value	0.72	*p*-value	1
Number of treatments received per patient, n (%)				
1	13 (31)	29 (69)	26 (62)	16 (38)
2	29 (49)	30 (51)	45 (76)	14 (24)
3	6 (43)	8 (57)	9 (64)	5 (36)
4	13 (35)	24 (65)	23 (62)	14 (38)
	p-value	0.27	*p*-value	0.36
Which lobes were treated per patient, n (%)				
right	33 (44)	42 (56)	54 (72)	21 (28)
left	10 (71)	4 (29)	12 (86)	2 (14)
both	18 (29)	45 (71)	37 (59)	26 (41)
		0.01		0.02
	Peri-interventional AEs (Day 1) n = 351 sessions		Acute AEs (Day 2 - Day 30) n = 351 sessions	
	0 AEs	≥1 AEs	0 AEs	≥1 AEs
Lobe treated per session, n (%)				
Right lobe	133 (56)	106 (44)	196 (82)	43 (18)
Left lobe	70 (63)	42 (37)	90 (80)	22 (20)
	p-value	0.32	*p*-value	0.78
Treatment session number, n (%)				
1	72 (47)	80 (53)	121 (80)	31 (20)
2	70 (64)	40 (36)	93 (85)	17 (15)
3	34 (66)	17 (33)	41 (80)	10 (20)
4	26 (70)	11 (30)	30 (81)	7 (19)
5	1 (100)	0 (0)	1 (100)	0 (0)
	p-value	0.01	*p*-value	0.86
Dose injected (categorized, 3 doses > 150), n (%)				
<=50 mg	26 (59)	18 (41)	30 (68)	14 (32)
<=100 mg	133 (54)	113 (46)	198 (80)	48 (20)
>100 mg	44 (72)	17 (28)	58 (95)	3 (5)
	p-value	0.04	*p*-value	0.002
Bead Size per session, n (%)				
100 µm	172 (64)	98 (36)	221 (82)	49 (18)
200 µm	31 (39)	49 (61)	64 (80)	16 (20)
400 µm	0 (0)	1 (100)	0 (0)	1 (100)
	p-value	<0.001	*p*-value	0.79
Treatment technically successful, n (%)				
No	199 (58)	147 (42)	281 (81)	65 (19)
Yes	4 (80)	1 (20)	5 (100)	0 (0)
	p-value	0.31	*p*-value	0.28

Supplementary Table S3.

**Procedural Medication Group as Defined in Figure 3**	**1**	**2**	**3**	**4**	**5**	**6**	***p* Value**
**Bead Size per session, n (%)**							
100	77 (62)	75 (88)	9 (60)	27 (82)	53 (96)	29 (76)	
200*	48 (38)	10 (12)	6 (40)	6 (18)	2 (4)	8 (21)	<0.001
400	0	0	0	0	0	1 (3)	
**Dose injected (categorized, 3 doses > 150), n (%)**							
<=50*	3 (2)	4 (5)	0	3 (9)	25 (45)	9 (24)	<0.001

<=100*	64 (51)	81 (95)	14 (93)	28 (85)	30 (55)	29 (76)	
>100	58 (46)	0	1 (7)	2 (6)	0	0	
**Percentage of liver involvement, n (%)**							
< 25%	54 (43)	42 (49)	5 (33)	21 (64)	40 (73)	26 (68)	
25 – 50%*	45 (36)	40 (47)	10 (67)	11 (33)	12 (22)	11 (29)	<0.001
> 50%*	26 (21)	3 (4)	0 (0)	1 (3)	3 (5)	1 (3)	

* Were found to be associated with patients who had at least 1 AE in our cohort.
